# Periodontal status and lung function decline in the community: the Hisayama study

**DOI:** 10.1038/s41598-018-31610-3

**Published:** 2018-09-06

**Authors:** Kenji Takeuchi, Koichiro Matsumoto, Michiko Furuta, Satoru Fukuyama, Toru Takeshita, Hiroaki Ogata, Shino Suma, Yukie Shibata, Yoshihiro Shimazaki, Jun Hata, Toshiharu Ninomiya, Yoichi Nakanishi, Hiromasa Inoue, Yoshihisa Yamashita

**Affiliations:** 1 Section of Preventive and Public Health Dentistry, Division of Oral Health, Growth and Development, Faculty of Dental Science, Kyushu University, Fukuoka, Japan; 2 OBT Research Center, Faculty of Dental Science, Kyushu University, Fukuoka, Japan; 3 Research Institute for Diseases of the Chest, Graduate School of Medical Sciences, Kyushu University, Fukuoka, Japan; 4 Department of Preventive Dentistry and Dental Public Health, School of Dentistry, Aichi Gakuin University, Aichi, Japan; 5 Department of Epidemiology and Public Health, Graduate School of Medical Sciences, Kyushu University, Fukuoka, Japan; 6 Center for Cohort Studies, Graduate School of Medical Sciences, Kyushu University, Fukuoka, Japan; 7 Department of Medicine and Clinical Science, Graduate School of Medical Sciences, Kyushu University, Fukuoka, Japan; 8 Department of Pulmonary Medicine, Graduate School of Medical and Dental Sciences, Kagoshima University, Kagoshima, Japan

## Abstract

This study aimed to determine whether periodontal status is related to a decline in lung function in a general Japanese population. We followed a total of 1,650 community-dwelling individuals (≥40 years) without chronic obstructive pulmonary disease, with at least one teeth, for 3 years. Periodontal status was assessed at baseline by clinical attachment loss (CAL) and probing pocket depth (PPD) at two sites for each tooth, and the mean values were calculated for each subject. Lung function was measured at baseline and follow-up using spirometry, and longitudinal decline in forced expiratory volume in one second (FEV_1_) was calculated. Multivariate Poisson regression with robust error variance was used to estimate risk ratio (RR). After adjusting for potential confounders including smoking status, there was a tendency for the adjusted RR of developing rapid lung function decline (≥160 mL/3years, the highest quartile of the distribution of FEV_1_ declines) to increase as mean CAL levels increased (*P* trend = 0.039). Likewise, a positive association was observed between mean PPD levels and RR of developing rapid lung function decline (*P* trend = 0.047). Our findings suggest deterioration of periodontal status could be a risk factor for rapid lung function decline in the general Japanese population.

## Introduction

Chronic obstructive pulmonary disease (COPD) is a substantial public health burden that results in approximately 3 million deaths annually worldwide^1,2^. Globally, COPD was rated the fourth leading cause of death in 2002 and the thirteenth leading cause of health burden overall, as measured by disability-adjusted life-years (DALYs). COPD is expected to rise to be the third leading cause of mortality and fifth leading cause of DALYs by 2030^3^. Further, the economic burden of COPD is considerable across countries, including Japan^4^, and will continue to grow as the number of older people continues to increase^5^. Longitudinally, COPD has been considered a consequence of rapid decline in lung function during adulthood, as assessed by forced expiratory volume in one second (FEV_1_)^6,7^. However, the causes of rapid lung function decline are not fully understood. Therefore, intensive research is needed to identify risk factors for rapid lung function decline, with obvious implications for preventive measures to decrease the burden of COPD on health systems.

Recently, a growing number of research studies have focused on the link between chronic inflammatory oral disease, such as periodontal disease, and impairment of lung function, including COPD^8–12^. Periodontal disease is a common chronic inflammatory disease affecting tissues that support the teeth. At least 40% of dentate adults aged ≥40 years in Japan experience periodontal disease^13^. Gingivitis, the mildest form of this disease, is highly prevalent worldwide, affecting 50–90% of the global population^14^. As periodontal disease and COPD are both chronic progressive inflammatory conditions or diseases, it has been proposed that they could be causally linked, sharing common pathophysiological processes. However, to our knowledge, no studies with a prospective cohort study design have revealed whether periodontal status is related to a decline in lung function parameters among healthy individuals. Therefore, we investigated whether periodontal status is related to decline in FEV_1_ over time by targeting a general adult population including older adults in Japan.

## Methods

### Study population

The present study was based on data from the Hisayama Study, an on-going population-based prospective cohort study of cardiovascular disease and its risk factors that were established in 1961 in the town of Hisayama, a suburb of the Fukuoka metropolitan area on Kyushu Island, Japan^15^. According to national census data, the distributions of age and occupation as well as the nutrient intake of the population of Hisayama have been almost identical to those across Japan during the past 50 years^16^.

Briefly, as a part of the study, 3-year follow-up cohort study was conducted among Hisayama residents from 2012 (baseline) to 2015 (follow-up). In 2012, 2,557 residents aged 40 years and older (55.3% of the total number of residents in this age group) consented to participate in a comprehensive oral and medical examination, including spirometry. After a 3-year follow-up (observation period, June 2012 to October 2015), 1,894 participants remained in the cohort (response rate, 74.1%). After excluding 135 participants with COPD at the baseline examination in 2012, 52 participants with asthma, 35 participants with no teeth, and 22 participants with missing responses to survey questions on other covariates used in the analysis, the remaining 1,650 participants (700 men, 950 women) formed the final population of this 3-year cohort study. Written informed consent was obtained from all participants. The Kyushu University Institutional Review Board for Clinical Research approved the study. All methods were performed in accordance with the approved guidelines and regulations.

### Measurement of lung function

Study participants underwent multiple spirometry tests with a minimum interval observation period of 3 years between the baseline and follow-up examinations. This 3-year period was considered necessary to obtain stable rates of FEV_1_ decline^17–19^. Spirometry was performed in accordance with guidelines of the Japanese Respiratory Society^20^ using a CHESTGRAPH HI-105 spirometer (Chest M.I., Inc., Tokyo, Japan), as described previously^21^. Measurements were taken among seated participants by specially trained laboratory technicians. At least three tests were conducted until satisfactory flow-volume curves were obtained. The results were assessed by two pulmonary physicians, who visually inspected the flow-volume curve and excluded participants without at least two satisfactory tests. The highest FEV_1_ and forced vital capacity (FVC) values were recorded. Reference values for FEV_1_% predicted were derived using Japanese criteria^20^. Participants who had pre-bronchodilator (BD) FEV_1_/FVC <70% were eligible for post-BD testing, in which spirometry was performed 15 minutes after inhalation of salbutamol (GlaxoSmithKline, Tokyo, Japan) via a metered-dose inhaler with a spacer, according to the recommended procedure^22^. We excluded from the primary analyses those participants with prevalent COPD GOLD (Global Initiative for Chronic Obstructive Lung Disease) stage I or greater (defined as post-BD FEV_1_/FVC <70%) or physician-diagnosed asthma at the baseline examination. Only data of pre-BD measurements were available for the entire cohort and were therefore used in the analyses.

**Figure 1:**
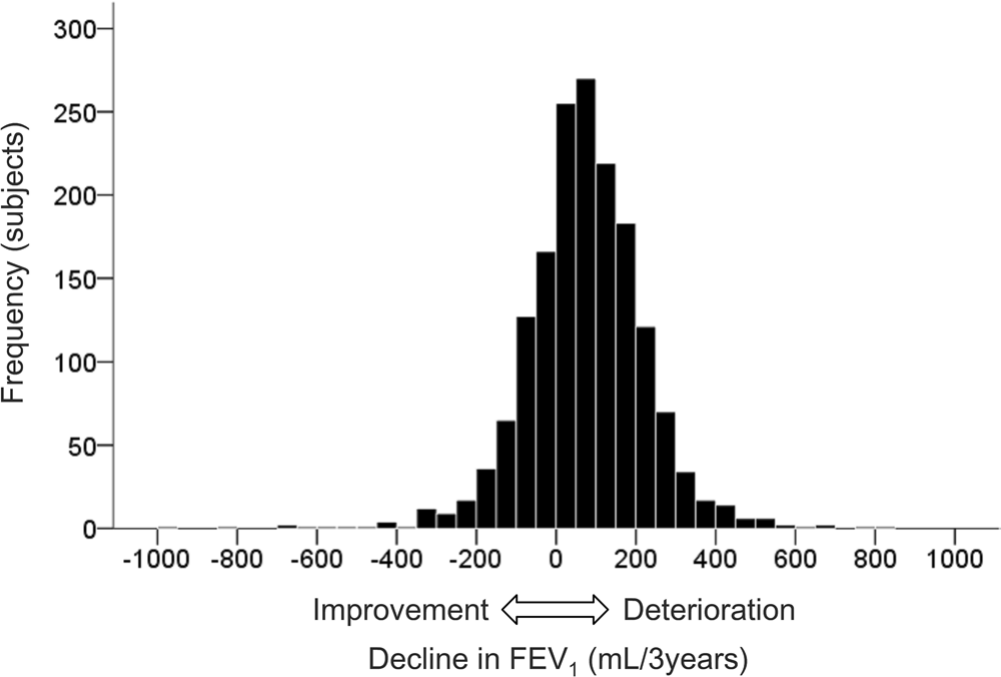
Distribution of the decline in forced expiratory volume in one second (FEV_1_), over a 3-year period.

The longitudinal absolute decline in FEV_1_ value was selected to reflect loss of lung function, as previously reported^17–19^. The decline in FEV_1_ was calculated by measuring the difference between the baseline and follow-up^7^. The calculated mean (±SE) decline in FEV_1_ over a 3-year period was 71 ± 4 mL/3years (Fig. 1). The FEV_1_ decline was classified into two groups as rapid decline (greater than 75th percentile, ≥160 mL/3years) and non-rapid decline (less than 75th percentile, <160 mL/3years), as defined in previous publications^19,23,24^. The primary outcome measurement was the development of rapid decline in FEV_1_.

### Measurement of periodontal status

At baseline, a clinical oral examination was performed by calibrated and licensed dentists, following the method of the Third National Health and Nutrition Examination Survey^25^. Training of the examiners and consensus discussions were conducted before initiation of the baseline examinations. Examiner reliability for the oral examination was verified by interexaminer calibration with volunteers who had characteristics similar to those of the study population, as previously described^26^. A manual periodontal probe was used (PCP11; Hu-Friedy Mfg. Co. LLC, Chicago, IL, USA). Periodontal status was assessed based on the clinical attachment loss (CAL) and probing pocket depth (PPD) at mesiobuccal and midbuccal sites for all present teeth, except for the third molars because partially impacted third molars frequently exhibit pseudo-pockets. CAL equals the distance from the cementoenamel junction to the pocket base and is used as the standardized measure of the severity of cumulative periodontal disease. PPD equals the distance from the free gingival margin to the pocket base and is used as the standardized measure of severity of current periodontal disease. Mean CAL and PPD over all measurement sites were calculated as the primary predictors of rapid FEV_1_ decline. Study participants were divided into four groups according to the quartile distribution of the mean CAL (first quartile: <1.46 mm, second quartile: 1.47–1.80 mm, third quartile: 1.81–2.23 mm, fourth quartile: ≥2.24 mm) and PPD (first quartile: <1.29 mm, second quartile: 1.30–1.62 mm, third quartile: 1.63–1.98 mm, fourth quartile: ≥1.99 mm).

### Measurement of other risk factors

We included a wide range of covariates in the analyses as potential confounding risk factors, based on the published literature. At the baseline examination, trained interviewers reviewed a self-administered questionnaire covering information on demographic characteristics, current occupation, medical history and treatment, physical activity, smoking status, and alcohol intake. Sex and age were queried as demographic characteristics. Occupation was used to stratify participants according to socioeconomic status, as follows: white-collar workers, blue-collar workers, unemployed, homemakers, and part-time workers^27^. A blood sample was collected from the antecubital vein in the morning after overnight fasting, and fasting levels of plasma glucose were determined using the hexokinase method. Glycated haemoglobin (HbA1c) levels were measured using latex aggregation immunoassay (Determiner HbA1C; Kyowa Medex, Tokyo, Japan) and were estimated as a National Glycohaemoglobin Standardization Program equivalent value. Diabetes mellitus was diagnosed by the American Diabetes Association criteria in 2003 as follows^28^: fasting plasma glucose level corresponding to 126 mg/dL (7.0 mmol/L), 2-hour postload or postprandial plasma glucose level corresponding to 200 mg/dL (11.1 mmol/L), or current treatment with insulin or oral hypoglycaemic medication. Body height and weight were measured with participants wearing light clothing and no shoes, and the body mass index (BMI) (kg/m^2^) was calculated. Physical activity status was defined as engaging in exercise at least one or more times per week during leisure time. Participants were divided into two groups according to level of physical activity, an active group and an inactive group. Smoking status was divided into smokers (current and ex-smokers) and never smokers. Next, intensity of smoking was classified by the Brinkman index (categorized as 0, 1–399, 400–799, or ≥800). The Brinkman index was an estimation of a lifetime tobacco consumption of each smoker, which was determined as the number of cigarettes per day multiplied by the number of years of smoking^29^. Alcohol intake was categorized as never, former, or current.

### Statistical analyses

Summary statistics for participant characteristics were constructed using percentages for categorical variables and mean ± SD for continuous variables. Linear trends in the percentages and the mean values of risk factors across mean CAL and PPD levels were tested using logistic regression analysis and linear regression analysis, respectively. To evaluate the relationship between periodontal status and lung function decline, we estimated crude and adjusted risk ratio (RR) respectively with 95% confidence interval (CI) for rapid decline in FEV_1_ based on baseline mean CAL and PPD levels, using Poisson regression with robust error variance. In the multivariate model, we included sex, age, occupation, diabetes mellitus, BMI, physical activity, Brinkman index, and alcohol intake as covariates. Additionally, because the decline in decline in FEV_1_ had a normal distribution, multiple linear regression models were even used to evaluate whether there was a similar association of mean CAL and mean PPD with the decline in FEV_1_ over a 3-year period. Two individual regression models were developed for each mean CAL and mean PPD levels with each adjusted for all covariates. All analyses were performed using IBM SPSS version 24 statistical software (IBM Corp., Armonk, NY, USA). Two-sided *P* values < 0.05 were considered statistically significant in all cases. We followed the STROBE statement guidelines for the analysis of observational data^30^.

## Results

The characteristics of the study population enrolled from 2012 to 2015 according to mean CAL and mean PPD levels are shown in Table 1 and Table 2. The participants consisted of 700 males and 950 females with an average age of 62.0 years. The percentages of men, participants having diabetes mellitus, and those with rapid declines in FEV_1_ increased gradually with higher mean CAL levels; mean values of age and BMI increased gradually with higher mean CAL levels; mean values of the FEV_1_% predicted and FEV_1_/FVC decreased gradually with higher mean CAL levels. The percentages of occupation types and Brinkman index distribution were significantly different across mean CAL levels. Likewise, the relevant association of mean PPD levels with the characteristics of the study population showed statistical significance (Table 2).

**Table 1:** Characteristics of the study participants according to quartile of mean CAL.

	Mean CAL				
	Q1 (Low) n = 414	Q2 n = 410	Q3 n = 410	Q4 (High) n = 416	*P* value
**At baseline examination**					
Men, %	26.1	40.5	43.2	59.9	<0.001
Age, years	58.0 ± 10.7	60.5 ± 10.4	63.0 ± 10.8	66.6 ± 10.9	<0.001
Occupation, %					
White-collar workers	33.1	31.0	31.2	26.2	0.045*
Blue-collar workers	14.0	14.1	17.1	21.4	
Unemployed, homemakers, and part-time workers	52.9	54.9	51.7	52.4	
Diabetes mellitus, %	10.9	12.0	16.3	22.8	<0.001
Body mass index	22.4 ± 3.5	23.0 ± 3.1	23.6 ± 3.4	23.7 ± 3.3	<0.001
Physically active, %	44.9	52.7	47.6	45.9	0.845
Brinkman index, %					
0 (Never smokers)	71.7	63.9	59.8	44.7	<0.001*
1–399 (Ex-smokers)	13.5	11.7	11.2	9.1	
400–799 (Ex-smokers)	4.1	7.3	9.3	11.8	
≥800 (Ex-smokers)	2.9	5.9	6.3	14.2	
1–399 (Current smokers)	2.7	3.7	3.2	3.6	
400–799 (Current smokers)	3.6	3.7	6.3	9.4	
≥800 (Current smokers)	1.4	3.9	3.9	7.2	
Alcohol intake, %					
Never	39.1	33.4	34.1	34.4	0.281*
Former	11.1	15.9	12.9	11.8	
Current	49.8	50.7	52.9	53.8	
FEV_1_, L	2.3 ± 0.6	2.4 ± 0.6	2.3 ± 0.6	2.3 ± 0.6	0.117
FEV_1_% predicted, %	96.9 ± 13.0	95.4 ± 12.7	95.3 ± 14.3	93.3 ± 15.0	<0.001
FEV_1_/FVC %, %	77.2 ± 5.1	77.2 ± 5.4	77.0 ± 5.5	75.7 ± 5.7	<0.001
**At follow-up examination**					
Decline in FEV_1_, mL/3years	56.7 ± 147.6	80.4 ± 156.4	71.7 ± 176.5	76.0 ± 152.5	0.158
Rapid decline in FEV_1_ (≥160 mL/3years), %	19.1	27.1	27.1	29.1	0.002

Quartiles for mean clinical attachment loss were <1.46, 1.47–1.80, 1.81–2.23, ≥2.24 mm.

CAL = clinical attachment loss; FEV_1_ = forced expiratory volume in one second; FVC = forced vital capacity.

Categorical variables were expressed as percentages.

Continuous variables were expressed as means ± SDs.

*Tested using chi-square test.

**Table 2:** Characteristics of the study participants according to quartile of mean PPD.

	Mean PPD				
	Q1 (Low) n = 412	Q2 n = 412	Q3 n = 412	Q4 (High) n = 414	*P* value
**At baseline examination**					
Men, %	31.8	38.3	43.4	56.0	<0.001
Age, years	59.7 ± 11.4	61.3 ± 10.4	62.1 ± 10.9	65.0 ± 11.3	<0.001
Occupation, %					
White-collar workers	33.7	31.6	27.9	28.3	0.001*
Blue-collar workers	12.1	13.3	18.4	22.7	
Unemployed, homemakers, and part-time workers	54.1	55.1	53.6	49.0	
Diabetes mellitus, %	10.4	14.1	16.3	21.3	<0.001
Body mass index	22.3 ± 3.3	23.1 ± 3.3	23.4 ± 3.4	23.9 ± 3.3	<0.001
Physically active, %	49.8	48.8	50.2	42.3	0.056
Brinkman index, %					
0 (Never smokers)	68.2	64.1	61.4	46.4	<0.001*
1–399 (Ex-smokers)	13.8	12.6	10.2	8.9	
400–799 (Ex-smokers)	5.3	8.3	7.0	11.8	
≥800 (Ex-smokers)	5.1	4.1	6.8	13.3	
1–399 (Current smokers)	2.7	3.4	4.1	2.9	
400–799 (Current smokers)	3.2	4.9	4.9	10.1	
≥800 (Current smokers)	1.7	2.7	5.6	6.5	
Alcohol intake, %					
Never	37.1	34.7	32.3	37.0	0.778*
Former	12.6	13.8	13.1	12.1	
Current	50.2	51.5	54.6	51.0	
FEV_1_, L	2.3 ± 0.6	2.3 ± 0.6	2.3 ± 0.6	2.3 ± 0.6	0.561
FEV_1_% predicted, %	96.5 ± 12.9	95.7 ± 14.6	96.0 ± 13.5	92.7 ± 14.1	<0.001
FEV_1_/FVC %, %	76.9 ± 5.5	77.2 ± 5.4	77.0 ± 5.1	76.0 ± 5.7	0.015
**At follow-up examination**					
Decline in FEV_1_, mL/3years	62.1 ± 144.4	72.8 ± 168.3	73.4 ± 160.6	76.5 ± 160.7	0.210
Rapid decline in FEV_1_ (≥160 mL/3years), %	21.6	25.5	24.5	30.7	0.006

Quartiles for mean probing pocket depth were <1.29, 1.30–1.62, 1.63–1.98, ≥1.99 mm.

PPD = probing pocket depth; FEV_1_ = forced expiratory volume in one second; FVC = forced vital capacity.

Categorical variables were expressed as percentages.

Continuous variables were expressed as means ± SDs.

*Tested using chi-square test.

The estimated RRs and 95% CIs of rapid decline in FEV_1_ according to mean CAL levels are shown in Table 3. The risk of developing a rapid decline in FEV_1_ significantly increased progressively with elevated mean CAL levels (*P* for trend = 0.001). This relationship remained significant after adjustment for potential confounders (*P* for trend = 0.039). The multivariable-adjusted RR of rapid decline in FEV_1_ was significantly higher among participants in the second, third, and fourth quartiles of mean CAL than among those in the first quartile (RR 1.32, 95% CI: 1.03–1.70 for the second quartile; RR 1.33, 95% CI: 1.03–1.72 for the third quartile; RR 1.35, 95% CI: 1.04–1.76 for the fourth quartile). When the RRs and 95% CIs of rapid decline in FEV_1_ were estimated considering mean PPD as periodontal exposure, the risk of developing a rapid decline in FEV_1_ increased progressively with elevated mean PPD levels in the univariate analysis (*P* for trend = 0.006), and this tendency was also statistically significant in the multivariable-adjusted analysis (*P* for trend = 0.047) (Table 4). In the multiple linear regression models, a one unit increase in mean CAL and mean PPD was associated with a 11.22 and 15.88 mL/3years increase in the decline in FEV_1_ over a 3-year period (mean CAL, *P* = 0.075; mean PPD, *P* = 0.071) (Table 5).

**Table 3:** Risk ratios for development of rapid decline in FEV_1_ according to quartile of mean CAL.

	Mean CAL				*P* for Trend
	Q1 (Low) n = 414	Q2 n = 410	Q3 n = 410	Q4 (High) n = 416	
Rapid decline in FEV_1_, n	79	111	111	121	
Crude RR (95% CI)	1.00 (reference)	1.42 (1.10–1.83)	1.42 (1.10–1.83)	1.52 (1.19–1.95)	0.001
Adjusted RR (95% CI)*	1.00 (reference)	1.32 (1.03–1.70)	1.33 (1.03–1.72)	1.35 (1.04–1.76)	0.039

Quartiles for mean clinical attachment loss were <1.46, 1.47–1.80, 1.81–2.23, ≥2.24 mm.

CAL = clinical attachment loss; FEV_1_ = forced expiratory volume in one second; RR = risk ratio; CI = confidence interval.

*Adjusted for sex, age, occupation, diabetes mellitus, body mass index, physical activity, Brinkman index, alcohol intake.

**Table 4:** Risk ratios for development of rapid decline in FEV_1_ according to quartile of mean PPD.

	Mean PPD				*P* for Trend
	Q1 (Low) n = 412	Q2 n = 412	Q3 n = 412	Q4 (High) n = 414	
Rapid decline in FEV_1_, n	89	105	101	127	
Crude RR (95% CI)	1.00 (reference)	1.18 (0.92–1.51)	1.13 (0.88–1.46)	1.42 (1.12–1.79)	0.006
Adjusted RR (95% CI)*	1.00 (reference)	1.16 (0.92–1.49)	1.10 (0.85–1.41)	1.33 (1.04–1.70)	0.047

Quartiles for mean probing pocket depth were <1.29, 1.30–1.62, 1.63–1.98, ≥1.99 mm.

PPD = probing pocket depth; FEV_1_ = forced expiratory volume in one second; RR = risk ratio; CI = confidence interval.

*Adjusted for sex, age, occupation, diabetes mellitus, body mass index, physical activity, Brinkman index, alcohol intake.

**Table 5:** Associations of mean CAL and mean PPD with the decline in FEV_1_ (mL/3years).

	*Β* (95% CI)	*P* value
Mean CAL, mm	11.22 (−1.13–23.57)	0.075
Mean PPD, mm	15.88 (−1.36–33.13)	0.071

CAL = clinical attachment loss; PPD = probing pocket depth; FEV_1_ = forced expiratory volume in one second; CI = confidence interval.

Models were adjusted for sex, age, occupation, diabetes mellitus, body mass index, physical activity, Brinkman index, alcohol intake.

## Discussion

In this prospective cohort study among a general population of Japanese adults, we demonstrated a clear relationship between periodontal status and risk of developing a rapid decline in FEV_1_, indicating that participants with higher mean CAL and PPD levels were at increased risk of onset of rapid FEV_1_ decline. This relationship was independent of smoking status and other important health characteristics. In addition, our results indicate that the longitudinal decline in FEV_1_ during the follow-up period had a tendency to increase as mean CAL and mean PPD increased. Therefore, it is reasonable to suppose that the optimal control of periodontal status is clinically important in reducing the risk of rapid lung function decline, which can in turn contribute to the prevention of future development of COPD.

Several studies have examined the association between indices of periodontal status and lung function parameters^9,31^. A large cross-sectional study in Germany reported that participants with higher CAL and PPD measurements had significantly lower values of FEV_1_^9^. Similarly, in a hospital-based cross-sectional study of patients with COPD in India, a significant negative correlation was observed between FEV_1_ values and CAL and PPD, thereby indicating a trend in which severity of impaired lung function increased as these indices of periodontal status worsened^31^. Our findings agree with those of these previous studies using a cross-sectional study design. Importantly, the present report, using a longitudinal study design, provides the first evidence supporting the hypothesis that periodontal status could be an important risk marker of rapid lung function decline in a general adult population.

There are two plausible pathways to explain the link between periodontal status and the development of rapid lung function decline. First, aspiration of potentially pathogenic oral contents has been known to play a role in airway inflammation under the circumstance that microaspiration is common even in healthy adults during sleep. The periodontal pocket provides an optimal microenvironment for bacterial growth, in other words, this is an important reservoir for potential respiratory pathogens. The levels of salivary periodontal pathogens have been reported to increase progressively with elevated pathogen burden in periodontal pockets^32^. Furthermore, periodontal tissues that are inflamed owing to oral bacteria secrete cytokines and biologically active substances. Thus, the aspiration of pathogenic bacteria, cytokines, neutrophils, and other biologically activated mediators into the lungs may lead to impaired respiratory function prior to the manifestation of COPD^33–35^. Another suggested pathway is circulating inflammatory mediators and bacteria^36^. Periodontal pathogens in periodontal pockets can access the gingival vasculature and permit the invasion of inflammatory mediators and bacteria into systemic circulation, with local inflammation of bronchial tissues^37^. Given that periodontal pathogens are present in periodontal pockets, mainly in the subgingival plaque, periodontal treatments such as subgingival curettage and periodontal flap surgery might be effective in reducing this plaque and the haematogenous dissemination of inflammatory mediators and bacteria^38^. Therefore, such treatment may help to reduce lung function decline.

The strengths of the present study are the prospective cohort design and large sample size with a broad age range (40–92 years) among a general Japanese adult population. In addition, two highly standardized periodontal exposure definitions, covering both cumulative and current periodontal status, were used. On the other hand, some potential limitations of the present study should be noted. A weakness of our study is that changes in the confounding factors during follow-up were not considered. The lack of this information may have impacted the accuracy of our findings to some extent. In addition, the partial recording methodology used in this study, such as a full-mouth periodontal examination with two sites per tooth, might underestimate the extent and severity of periodontal status. However, an association found even with the underestimation of periodontal progression will be likely more robust when a more complete estimation, such as a full-mouth examination with six sites per tooth, is used^39^.

In conclusion, the present study demonstrated that deterioration of periodontal status could be a significant risk factor for the development of rapid lung function decline in a general population of Japanese adults. Our findings suggest that promoting and supporting opportunities for oral care and treatment, especially in terms of maintenance of periodontal health, might be an effective strategy for reducing the burden of lung function impairment leading to COPD.

## Data Availability

All data used are from the Hisayama study. The Hisayaama study data used in this study will be made available upon request due to ethical restrictions. Interested researchers must be approved by the Kyushu University Institutional Review Board for Clinical Research. Thus, to request the data, please contact Dr. Yoshihisa Yamashita, Section of Preventive and Public Health Dentistry, Division of Oral Health, Growth and Development, Faculty of Dental Science, Kyushu University via email: yoshi@dent.kyushu-u.ac.jp. All Hisayama study datasets have ethical or legal restrictions for public deposition due to inclusion of sensitive information from the human participants.
